# Effectiveness of Teriparatide on Fracture Healing: A Systematic Review and Meta-Analysis

**DOI:** 10.1371/journal.pone.0168691

**Published:** 2016-12-20

**Authors:** Zhongju Shi, Hengxing Zhou, Bin Pan, Lu Lu, Jun Liu, Yi Kang, Xue Yao, Shiqing Feng

**Affiliations:** Department of Orthopaedics, Tianjin Medical University General Hospital, Tianjin, PR China; Medical University Innsbruck, AUSTRIA

## Abstract

**Purpose:**

Nowadays, the efficacy of teriparatide in treating osteoporosis was widely accepted, but the discussion about using teriparatide to enhance fracture healing hasn’t come to an agreement. This meta-analysis was conducted to evaluate the effectiveness of teriparatide for fracture healing.

**Methods:**

We searched PubMed, the Cochrane Library, and Embase in August 2016 for randomized controlled trials (RCTs) which concerned the treatment of teriparatide for fracture healing.

**Results:**

Finally, a total of 380 patients were randomly assigned in the 5 trials included in this meta-analysis. There was a significant effectiveness with regards to function improvement in patients following fracture, however, there was no significant effectiveness with regards to time of radiographic fracture healing, fracture healing rate and reduction in pain.

**Conclusions:**

This analysis showed that administration of teriparatide following fracture lacked the effectiveness for fracture healing. Moreover, teriparatide administration had no apparent adverse effects. These results should be interpreted with caution because of some clear limitations. If we want to confirm whether teriparatide improves fracture healing, more high-quality randomized controlled trials are needed.

## Introduction

Fractures are the most common large-organ, trauma to humans [1]. It is estimated that approximately 7.9 million fractures are sustained in the United States annually [2]. Approximately 10% of fractures can occur as fracture nonunion, and many others result in compromised regeneration such as delayed union or improper tissue restoration [3]. Fracture healing contains an anabolic phase and prolonged catabolic phase, and the former can increase tissue volume and form new skeletal tissues, while the latter can recover to the original structure [1,4,5]. Therefore, understanding of bone loss and repair and identifying strategies to promote fracture healing are essential for the clinical treatment of fractures.

Parathyroid hormone (PTH) is a major research topic in increasing bone mass, and it is the only anabolic bone therapeutic option approved by the Food and Drug Administration (FDA) for patients with low bone mass conditions, such as osteoporosis, to date [6–9]. The intact hormone PTH (1–84) and its N-terminal fragment (1–34) have been used to treat established osteoporosis [10]. Teriparatide is approved and plays an important role in treating osteoporosis and glucocorticoid-induced osteoporosis in patients at a high fracture risk of fractures [11]. Previous studies also showed that systemic administration of teriparatide could cause a rapid increase in bone formation biomarkers and a delay bone resorption biomarkers [12,13]. Several studies have shown that teriparatide can enhance fracture healing. Among these, some animal studies have shown that daily systemic administration of PTH (1–34) increased bone mineral content, density and strength to improve fracture healing, and it can produce a sustained anabolic effect throughout the remodelling phase of fracture healing [14,15]. Several case reports have also suggested that teriparatide can accelerate fracture healing for patients with fracture at different skeletal sites [16–18].

To determine whether teriparatide improves fracture healing, we performed a systematic literature review and meta-analysis of randomized controlled trials (RCTs). In this study, we analysed the effect of teriparatide on the time of fracture healing, fracture healing rate, pain and functional recovery.

## Materials and Methods

### Search strategy

We searched PubMed, the Cochrane Library, and Embase in August 2016 for studies using the following combination of terms: “Human Parathyroid Hormone (1–34)”, “hPTH (1–34)”, “Parathyroid Hormone 1–84”, “Parathar”, “Aventis Brand of Teriparatide”, “Teriparatide Aventis Brand”, “Teriparatide Acetate”,” Forteo”,” Lilly Brand of Teriparatide” and “Teriparatide Lilly Brand” in combination with “Fracture Healings”,” Healing, Fracture” and “Healings, Fracture”, no language restrictions were applied. The search strategy was shown in S1 File. Reference lists of the selected papers were also reviewed. Two reviewers independently searched and assessed all titles and abstracts and then screened the full texts of potentially relevant studies for final inclusion. We did not contact authors of the primary studies for additional information. Only complete original journal articles were included.

### Study selection

Studies were considered for inclusion if they met the following criteria: (1) the type of study design was a RCT; (2) participants were adults with acute fractures and were treated with teriparatide following fracture; (3) the teriparatide intervention was compared with placebo treatment at the same time, no therapy or comparator interventions.

Studies were excluded if they met the following criteria: (1) participants previously used teriparatide or parathyroid hormone, unless patients had undergone a wash-out period; (2) contraindication to any of the study drugs, formerly or currently on any of them; (3) serum calcium above the reference level and liver enzymes more than double of the upper limit; (4) non-RCTs or studies published as the following article type: abstracts, review articles and letters.

### Data extraction

The information which we extracted were as follows: (1) name of first author, publication year; (2) participants’ characteristics; (3) type of fracture and treatment; (4) number of cases; (5) follow-up; (6) time of radiological fracture healing, fracture healing rate, pain score and functional outcome from each study.

The primary endpoints were the time of fracture healing and fracture healing rate, as determined by radiography, which was defined as the time of cortical bridging by the trabecular or osseous bone in three of four cortices [19–22]. Pain score was assessed with numeric visual analog scale (VAS) score and “Patient-Rated Wrist Evaluation” (PRWE) pain score [20,23,22]. The functional outcome was defined as an improvement in mobility following treatment, and it’s assessed with the Timed “Up and Go” (TUG) test or the “Patient-Rated Wrist Evaluation” (PRWE) questionnaire or “disabilities of the arm, shoulder, and hand” (DASH) score [20,23,22].

### Statistical analysis

Data were analyzed using Review Manager Software (RevMan version 5.2; The Nordic Cochrane Center, The Cochrane Collaboration, Copenhagen, Denmark). The results were expressed in terms of odds ratio (OR) and a 95% confidence interval (CI) for dichotomous outcomes and in terms of mean difference (MD) and 95% CI for continuous outcomes. *P* < 0.05 was considered statistically significant. We used Cochran’s Q statistic, I^2^ statistic (I^2^ > 50% was used as a threshold indicating significant heterogeneity), and *P* values (*P* value < 0.10 was used as a threshold indicating significant heterogeneity) to assess the heterogeneity [24]. I^2^ was the proportion of total variation observed between the trials attributable to differences between trials. A random effects model was applied in the meta-analysis. [25]. Sensitivity analyses were also conducted to evaluate stability and heterogeneity of the results. This study conformed to Preferred Reporting Items for Systematic Reviews and Meta-Analyses (PRISMA) guidelines (S2 File).

## Results

### Literature search

**Fig 1:**
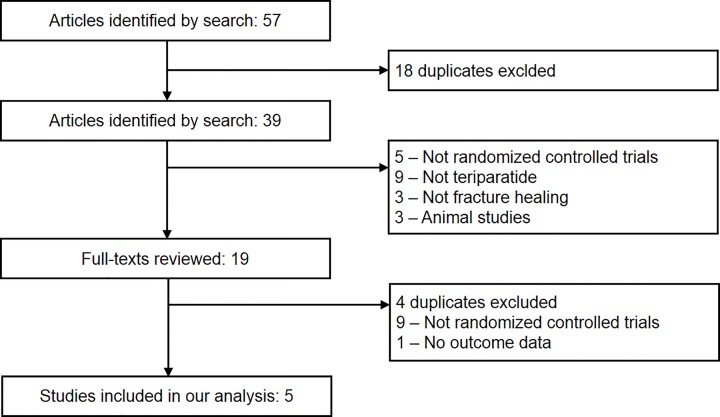
Flow diagram of selection of studies.

The literature search identified 57 trials until August 2016, most were excluded because they were duplicate, non-RCTs or because the exposure or endpoint was not relevant to our analysis. After assessing the full-text, five trials were finally designed to evaluate the effect of teriparatide on fracture healing. Fig 1 shows the process of study selection.

### Patient characteristics

A total of 380 patients were randomly assigned in the 5 trials included in this meta-analysis. Regarding sex, 11.1% (n = 42) of patients were men and 88.9% (n = 338) were women. Fracture types include distal radius fracture, femoral neck fracture, proximal humeral fracture, lower-extremity stress fracture and pelvic fracture. The overall mean age was 57.9 years. The more detailed characteristics of the included studies are listed in Table 1.

**Table 1:** Characteristics of included studies.

Study ID	Number of patients	Age/years		Sex		Type of fracture
		Mean	SD	Male	Female	
Aspenberg 2010	102	61.4	8.6	0	102	Distal radius fracture
Bhandari 2016	159	45.5	10	42	117	Femoral neck fracture
Johansson 2016	40	68	8.6	0	40	Proximal humeral fracture
Almirol 2016	14	31.4	4.4	0	14	Lower-extremity stress fracture
Peichl 2011	65	82.3	4.1	0	65	Pelvic fracture

SD, standard deviation.

### Trial design

In five trials, patients were randomly assigned to teriparatide in addition to standard-of-care therapy. The schedule varied as shown in Table 2. In the experimental group, the interventions included teriparatide and PTH (1–84). Teriparatide can be injected subcutaneously as a single dose of 20 μg or 40 μg every day. PTH (1–84) can be given at a concentration of 100 μg per day, and the anabolic effect of 100 μg PTH (1–84) is equal to 20 μg teriparatide because of the differences in pharmacokinetics and actions between these two types of PTH [26]. The interventional time of the teriparatide varied from 4 weeks to 24 months. The control group included the placebo instead of teriparatide or no therapy. In one trial, calcium and vitamin D were taken in the experimental group and control group.

**Table 2:** Detail of intervention.

Study ID	Intervention		N_e_/ N_c_	Treatment time	Time of initiation
	Experimental group	Control group			
Aspenberg 2010	Teriparatide 20 μg/day or 40 μg/day	Placebo	68/34	8 weeks	<10 days
Bhandari 2016	Teriparatide 20 μg/day	Placebo	78/81	6 months	<7 day
Johansson 2016	Teriparatide 20 μg/day	No therapy	20/20	4 weeks	<10 days
Almirol 2016	Teriparatide 20 μg/day	Placebo	6/8	8 weeks	<4 weeks
Peichl 2011	PTH (1–84) 100 μg/day, calcium 1000mg, vitamin D 800 IU	Calcium 1000mg, vitamin D 800 IU	21/44	24 months	<2 days

N_e_, number of patients in experimental group; N_c_, number of patients in control group.

### Quality of trials

**Fig 2:**
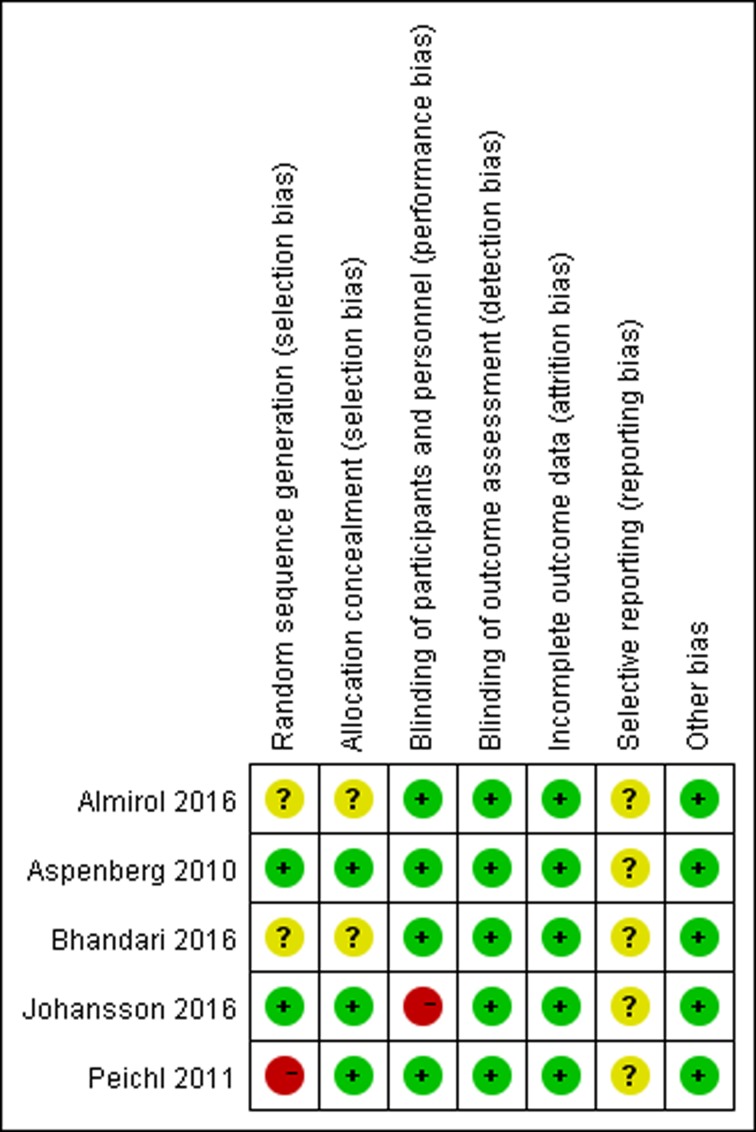
Risk of bias summary. The red with a minus means high risk of bias; the yellow with a question mark means unclear; the green with a plus means low risk of bias.

The quality assessment of the included trials has been performed according to the cochrane risk of bias tool, as described in detail in Fig 2. There were two RCTs were at low risk of bias for sequence generation, and two were at unclear risk and one was at high risk. All the RCTs were at low risk for allocation concealment. Only one trial was at high risk of blinding of participants and personnel, while other trials were at low risk of blinding. We appraised the rate of patients lost to follow-up, and there were three trials reporting losses to follow-up, but the rate were all lower than 20%.

### Radiographic assessment of fracture healing

**Fig 3:**
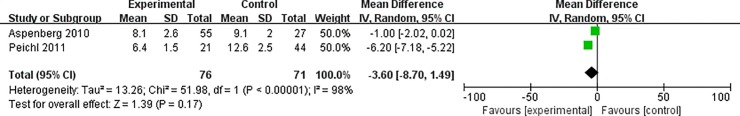
Forest plot for radiological fracture healing time.

Time of radiographic fracture healing was defined as the time to fracture bridging by trabecular or osseous bone in three of four cortices as seen on either anteroposterior or lateral radiographs. Two trials (147 patients) were eligible for the meta-analysis of radiological fracture healing times. According to the results, patients who were treated with early teriparatide therapy had no statistically significant difference in radiological fracture healing times compared with patients in the control group (MD -3.60, 95% CI -8.70 to 1.49; I^2^ of heterogeneity 98%, P<0.00001; random effects model) (Fig 3). The result indicated significant heterogeneity, but we didn’t performed a sensitivity analysis conditioned to the number of trials, and we hypothesized that the age maybe the source resulting in the heterogeneity.

**Fig 4:**
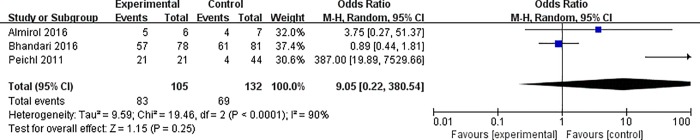
Forest plot for radiological fracture healing rate.

Then we also analysed the fracture healing rate, and three trials (237 patients) were eligible for the meta-analysis of fracture healing rate following intervention. According to the results, patients who were treated with teriparatide therapy had no statistically significant difference in fracture healing rate compared with the patients in the control group (OR 9.05, 95% CI 0.22 to 380.5; I^2^ of heterogeneity 90%, *P*<0.00001; random effects model) (Fig 4). As I^2^ = 90%, apparently over 50%, indicated significant heterogeneity, we further performed a sensitivity analysis and found that one trial significantly affected the OR (Table 3).

**Table 3:** Sensitivity analyses based on various exclusion criteria for fracture healing rate.

Number of excluded trial	Number of trials	Number of patients	Experimental group	Control group	OR (95% CI)	*P* value for OR	I^2^, %	*P* value for heterogeneity
[19]	2	224	99	125	16.11 [0.02, 12740.83]	0.41	95	<0.0001
[21]	2	79	27	51	36.11 [0.36, 3601.30]	0.13	82	0.02
[22]	2	173	84	88	1.03 [0.44, 2.41]	0.95	8	0.30

OR, odds ratio.

### Pain

Reduction in pain is also an important indicator for evaluating the fracture healing. VAS score and PRWE pain score were two common methods to evaluate pain degree and they were used in three trials [20,23,22]. VAS score was used to assess pain and numbness, and PRWE was applied to assess pain and function in patients with distal radius fracture [27,28].

**Fig 5:**
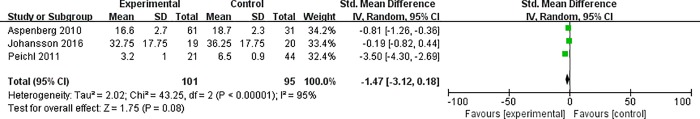
Forest plot for radiological fracture pain degree.

Three trials (196 patients) were eligible for the meta-analysis of VAS following intervention. Because of the different range and scoring methods, a standardized mean difference (SMD) method was used. According to the results, patients who were treated with teriparatide had no statistically significant difference in pain score compared with the patients in the control group (SMD -1.47, 95% CI -3.12 to 0.18; I^2^ of heterogeneity 95%, *P*<0.00001; random effects model) (Fig 5). As I^2^ = 95%, apparently over 50%, indicated significant heterogeneity, we further performed a sensitivity analysis and found that one trial significantly affected the SMD, and the heterogeneity could lower when removing the trial Peichl 2011 (Table 4).

**Table 4:** Sensitivity analyses based on various exclusion criteria for pain degree.

Number of excluded trial	Number of trials	Number of patients	Experimental group	Control group	SMD (95% CI)	*P* value for SMD	I^2^, %	*P* value for heterogeneity
[20]	2	104	40	64	-1.83 [-5.07, 1.40]	0.27	97	<0.00001
[23]	2	157	82	75	-2.13 [-4.76, 0.50]	0.11	97	<0.00001
[22]	2	131	80	51	-0.54 [-1.14, 0.06]	0.08	59	0.12

SMD, standardized mean difference.

### Functional outcome

**Fig 6:**
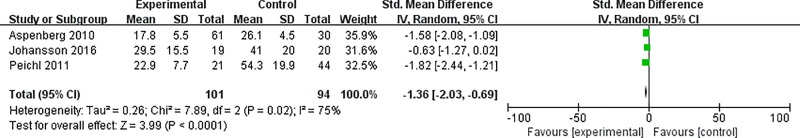
Forest plot for radiological fracture functional outcome.

The methods of evaluating the functional outcome include PRWE score, DASH and TUG test, and they can be used in different types of fractures. Because of the different measurement methods, a standardized mean difference method was used. Patients who were treated with teriparatide showed significantly better functional outcome than those in the control group (SMD -1.36, 95% CI -2.03 to 0.69; I^2^ of heterogeneity 75%, *P* = 0.02; random effects model) (Fig 6). As I^2^ = 75%, apparently over 50%, indicated significant heterogeneity, we further performed a sensitivity analysis and the heterogeneity could lower when removing the trial Johansson 2016 (Table 5).

**Table 5:** Sensitivity analyses based on various exclusion criteria for functional outcome.

Number of excluded trial	Number of trials	Number of patients	Experimental group	Control group	SMD (95% CI)	*P* value for SMD	I^2^, %	*P* value for heterogeneity
[20]	2	104	40	64	-1.23 [-2.40, -0.06]	0.04	86	0.008
[23]	2	157	82	74	-1.68 [-2.07, -1.29]	<0.00001	0	0.55
[22]	2	131	80	50	-1.13 [-2.07, -0.19]	0.02	81	0.02

SMD, standardized mean difference.

### Adverse events

Significant differences were identified between the experimental group and the control group with regard to slight bruising at the injection site, and there was no statistically significant difference between the experimental group and the control group regarding nausea, sweating, hypercalcemia, and headache (Table 6).

**Table 6:** Adverse effects.

Study ID	Adverse effects	Time	Rate of experimental group n,%	Rate of control group n,%	*P* value
Aspenberg 2010	Serious adverse events	-	0(0)	3(8.8%)	0.046
	Hypercalcemia	-	0(0)	1	0.490
	Nausea	-	7(40 mg group)	0(0)	0.279
	A new distal radius fracture	-	0(0)	1	0.490
Bhandari 2016	Patients with ≥ 1 adverse events	-	35 (45%)	40 (49%)	0.634
	Patients with ≥ 1 adverse events possibly related to study drug	-	5 (6%)	5 (6%)	1.000
	Patients with ≥ 1 serious adverse events	-	3 (4%)	7 (9%)	0.329
Johansson 2016	Nausea	First 1–5 days	0(0)	3(15.8%)	0.160
	Episodes of sweating	-	0(0)	2(10.5%)	0.260
	Slight headache	-	0(0)	1(5.3%)	0.470
Almirol 2016	Slight bruising at the injection site	-	6(100%)	0(0)	0.010
	Pea-sized bump below the site of fracture	-	1(16.7%)	0(0)	0.410
	Light-headedness	-	0(0)	1(14.3)	0.520
Peichl 2011	-	-	0(0)	0(0)	-

## Discussion

Currently, the efficacy of teriparatide in treating osteoporosis has been widely accepted, yet using teriparatide to enhance fracture healing is still debated. To our knowledge, this is not the first meta-analysis to examine the effect of teriparatide on fracture healing [29]. This previous study only included the osteoporotic patients, and outcomes in this study included radiological fracture healing time and functional outcome. However, in our study, the included patients were more than osteoporotic patients, and we also analysed some other outcomes of interest included fracture healing rate and reduction in pain. Moreover, recently published primary studies were also included in our meta-analysis. In this meta-analysis, which included 380 patients with fracture from five RCTs, we summarized the five studies in efficacy and safety of systemic administration of teriparatide and the analysis showed that teriparatide lacked the effectiveness for fracture healing in patients following fracture. Moreover, teriparatide administration had no apparent adverse effects.

Fracture healing is a complex process involving interaction of cellular elements and formation of new bone [30,31]. Delayed union or nonunion of fractures is a difficult problem that affects prognosis in the treatment of fracture; delayed union is defined when the fracture fails to unite within 6 months and nonunion when union has failed within 9 months [30,32]. Teriparatide can enhance the active building of bone mass via stimulation of the proliferation and differentiation of osteoprogenitor cells and it’s approved by the FDA in 2002 [33,34]. Teriparatide has been shown to promote fracture healing in various animal models [14,35,15]. Because of the anabolic effects of teriparatide on bone and its demonstrated efficacy for decreasing fracture risk, we hypothesized that teriparatide may promote fracture healing as well [36–38]. Therefore, we believed a systemic review and meta-analysis is required to evaluate the efficacy and safety of teriparatide for fracture healing.

In a related meta-analysis, the result showed that teriparatide was effective in promoting fracture healing and improving functional outcome of osteoporotic women, yet the evidence was not conclusive because of several limitations [29]. In our study, we conducted a comprehensive search for RCTs which compared the teriparatide intervention with placebo treatment at the same time, then we did a meta-analysis. The results showed that teriparatide could significantly improve function improvement in patients following fracture, and this result was consistent with the related study [29]. However, there was no significant effectiveness with regards to time of radiographic fracture healing, fracture healing rate and reduction in pain. Among the above indicators, fracture healing rate and time of radiographic fracture healing had important clinical value in the assessment of fracture healing. Therefore, these results showed a lack of effectiveness of teriparatide on fracture healing. This was not consistent with the previous study [29], possible reasons may be that the previous study only included osteoporotic women and recently published primary studies were included in our study.

Moreover, in this meta-analysis, the results showed significant heterogeneity, and then we performed sensitivity analysis and found the trials significantly affected the heterogeneity. The significant heterogeneity may be mainly because interventions in our meta-analysis varied greatly with respect to intervention type and intervention time. One possible explanation was the differences in the treatment time (4 weeks to 24 months) among the included studies, and different treatment times may have different therapeutic effects, so a uniform standard for treatment time was needed to improve the scientific evidence base and support clinical decision making. The other alternative explanation might be related to the age of the patients, which may has an effect on fracture healing rate and time of radiographic fracture healing. Future efforts should be made to perform high-quality RCTs with normative intervention of teriparatide.

Furthermore, some limitations of this meta-analysis should be considered. The sample sizes of most of the included studies and the study number included in this analysis were small, which could be a possible reason for detecting no statistically significant differences. Another limitation was the diversity of the control groups, and the included trials contained three types of control groups: those receiving placebo in parallel, those receiving calcium and vitamin D, or those receiving no therapy. The treatment time ranged from 4 weeks to 24 months, and the time of initiation were also different in these five studies. Therefore, the consistency of the intervention was difficult to guarantee. Additionally, as more than 88.9% (n = 338) of fractures occurred in females and only 11.1% (n = 42) of the patients were males, the results of the analysis were more applicable to females. Thus, these limitations lead to an insufficient evidence and more high-quality randomized controlled trials are needed.

## Conclusion

In conclusion, our findings suggested that administration of teriparatide following fracture lacked the effectiveness for fracture healing. We believed that these results were limited by the inadequacy of the studies, so these results should be interpreted with caution. If we want to confirm whether teriparatide improves fracture healing, more high-quality randomized controlled trials are needed.

## Supporting Information

S1 File Search strategies for databases. (DOCX)

S2 File PRISMA 2009 checklist. (DOC)
