# Supplementary material for: Effectiveness of Teriparatide on Fracture Healing: A Systematic Review and Meta-Analysis
Source: PLoS One. 2016 Dec 20;11(12):e0168691. doi: 10.1371/journal.pone.0168691 (PMC5173248; doi:10.1371/journal.pone.0168691)
Supplement: S1 File — (DOCX) [file pone.0168691.s001.docx]

**S1 File. Search strategies for databases.**

**PUBMED**

((((("Fracture Healing"[Mesh]) OR (((Fracture Healings[Title/Abstract]) OR Healing, Fracture[Title/Abstract]) OR Healings, Fracture[Title/Abstract])))) AND (((((((((((((Human Parathyroid Hormone (1-34)[Title/Abstract]) OR hPTH (1-34)[Title/Abstract]) OR Parathar[Title/Abstract]) OR Aventis Brand of Teriparatide[Title/Abstract]) OR Teriparatide Aventis Brand[Title/Abstract]) OR Teriparatide Acetate[Title/Abstract]) OR Forteo[Title/Abstract]) OR Lilly Brand of Teriparatide[Title/Abstract]) OR Teriparatide Lilly Brand[Title/Abstract])) OR "Teriparatide"[Mesh])) OR Parathyroid Hormone 1-84[Title/Abstract])) AND (randomized controlled trial[Publication Type] OR randomized[Title/Abstract] OR placebo[Title/Abstract])

**Embase**

#8. 'parathyroid hormone[1-34]'/exp OR

'teriparatide':ab,ti OR 'human parathyroid

hormone (1-34)':ab,ti OR 'hpth (1-34)':ab,ti OR

'parathar':ab,ti OR 'aventis brand of

teriparatide':ab,ti OR 'teriparatide aventis

brand':ab,ti OR 'teriparatide acetate':ab,ti OR

'forteo':ab,ti OR 'lilly brand of

teriparatide':ab,ti OR 'teriparatide lilly

brand':ab,ti OR 'parathyroid hormone 1-84':ab,ti

AND ('fracture healing'/exp OR 'fracture

healings':ab,ti OR 'healing, fracture':ab,ti OR

'healings, fracture':ab,ti OR

'fracture-healing':ab,ti) AND 'randomized

controlled trial'/exp

#7. 'randomized controlled trial'/exp

#6. 'fracture healing'/exp OR 'fracture

healings':ab,ti OR 'healing, fracture':ab,ti OR

'healings, fracture':ab,ti OR

'fracture-healing':ab,ti

#5. 'fracture healings':ab,ti OR 'healing,

fracture':ab,ti OR 'healings, fracture':ab,ti OR

'fracture-healing':ab,ti

#4. 'fracture healing'/exp

#3. 'parathyroid hormone[1-34]'/exp OR

'teriparatide':ab,ti OR 'human parathyroid

hormone (1-34)':ab,ti OR 'hpth (1-34)':ab,ti OR

'parathar':ab,ti OR 'aventis brand of

teriparatide':ab,ti OR 'teriparatide aventis

brand':ab,ti OR 'teriparatide acetate':ab,ti OR

'forteo':ab,ti OR 'lilly brand of

teriparatide':ab,ti OR 'teriparatide lilly

brand':ab,ti OR 'parathyroid hormone 1-84':ab,ti

#2. 'teriparatide':ab,ti OR 'human parathyroid

hormone (1-34)':ab,ti OR 'hpth (1-34)':ab,ti OR

'parathar':ab,ti OR 'aventis brand of

teriparatide':ab,ti OR 'teriparatide aventis

brand':ab,ti OR 'teriparatide acetate':ab,ti OR

'forteo':ab,ti OR 'lilly brand of

teriparatide':ab,ti OR 'teriparatide lilly

brand':ab,ti OR 'parathyroid hormone 1-84':ab,ti

#1. 'parathyroid hormone[1-34]'/exp

**Cochrane Library**

ID Search

#1 MeSH descriptor: [Teriparatide] explode all trees

#2 Human Parathyroid Hormone (1-34):ti,ab,kw or hPTH (1-34):ti,ab,kw or Parathar:ti,ab,kw or Aventis Brand of Teriparatide:ti,ab,kw or Teriparatide Aventis Brand:ti,ab,kw or Parathyroid Hormone 1-84:ti,ab,kw (Word variations have been searched)

#3 Teriparatide Acetate:ti,ab,kw or Forteo:ti,ab,kw or Lilly Brand of Teriparatide:ti,ab,kw or Teriparatide Lilly Brand:ti,ab,kw (Word variations have been searched)

#4 #1 or #2 or #3

#5 MeSH descriptor: [Fracture Healing] explode all trees

#6 Fracture Healings:ti,ab,kw or Healing, Fracture:ti,ab,kw or Healings, Fracture:ti,ab,kw (Word variations have been searched)

#7 #5 or #6

#8 #4 and #7
